# From Gas Phase Observations to Solid State Reality: The Identification and Isolation of Trinuclear Salicylaldoximato Copper Complexes

**DOI:** 10.3390/molecules27196421

**Published:** 2022-09-29

**Authors:** Benjamin D. Roach, Ross S. Forgan, Eduardo Kamenetzky, Simon Parsons, Paul G. Plieger, Fraser J. White, Sidney Woodhouse, Peter A. Tasker

**Affiliations:** 1 EaStCHEM School of Chemistry, Edinburgh EH9 3FJ, UK; 2 Solvay Industries, 1937 West Main Street, Stamford, CT 06904-0060, USA; 3 School of Natural Sciences, Massey University, Private Bag 11 222, Palmerston North 4410, New Zealand

**Keywords:** Cu extraction, Electrospray mass spectrometry, solvent extraction, trinuclear complexes, oxime proligands

## Abstract

Conditions have been identified in which phenolic aldoximes and ketoximes of the types used in commercial solvent extraction processes can be doubly deprotonated and generate polynuclear Cu complexes with lower extractant:Cu molar ratios than those found in commercial operations. Electrospray mass spectrometry has provided an insight into the solution speciation in extraction experiments and has identified conditions to allow isolation and characterization of polynuclear Cu-complexes. Elevation of pH is effective in enhancing the formation of trinuclear complexes containing planar {Cu_3_-μ_3_-O}^4+^ or {Cu_3_-μ_3_-OH}^5+^ units. DFT calculations suggest that such trinuclear complexes are more stable than other polynuclear species. Solid structures of complexes formed by a salicylaldoxime with a piperidino substituent *ortho* to the phenolic OH group (**L^9^**H_2_) contain two trinuclear units in a supramolecular assembly, {[Cu_3_OH(**L^9^**H)_3_(ClO_4_)](ClO_4_)} _2_, formed by H-bonding between the central {Cu_3_-μ_3_-OH}^5+^ units and oxygen atoms in the ligands of an adjacent complex. Whilst the lower ligand:Cu molar ratios provide more efficient Cu-loading in solvent extraction processes, the requirement to raise the pH of the aqueous phase to achieve this will make it impractical in most commercial operations because extraction will be accompanied by the precipitation (as oxyhydroxides) of Fe(III) which is present in significant quantities in feed solutions generated by acid leaching of most Cu ores.

## 1. Introduction

**Figure 1:**
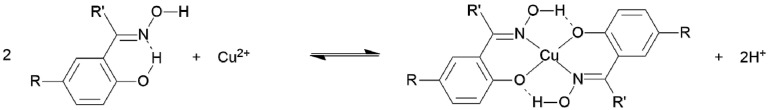
The pH-dependent extraction of Cu(II) by phenolic oximes.

Phenolic oximes are used extensively in the recovery of copper by hydrometallurgical processes which accounted for approximately 20% of worldwide production in 2020 [1,2,3]. The efficient mining and processing of Cu is of increasing importance for green technologies to achieve 2050 net zero goals, from renewable energy to electric vehicles. The separation and concentration of Cu(II) from aqueous sulfate solutions which usually contain an excess of Fe(III) is currently accomplished by highly selective solvent extraction using the reaction shown in Equation (1). The loading and stripping mechanisms of Cu(II) are pH-dependent as the phenolic hydroxy group is deprotonated in the charge-neutral complexes formed in the water-immiscible phase, usually a hydrocarbon with a high boiling point such as kerosene [4,5,6] (see Figure 1).

2LH_2(org)_ + Cu^2+^ = [Cu(LH)_2_]_(org)_ + 2H^+^ (1)

Deprotonation of both hydroxyl groups is also possible in phenolic oximes, often leading to the formation of polynuclear complexes, particularly with higher oxidation state metal ions, for example, Mn [7,8,9,10,11,12,13,14,15] and Fe [7,14,16,17,18,19,20].

In the context of copper recovery, extraction by doubly deprotonated (dianionic) forms of the phenolic oxime reagents offers the possibility of doubling the mass transport efficiency, as demonstrated in Equation (2), because the molar ratio of extractant to copper is 1:1 as opposed to 2:1. This paper considers the design features of phenolic oxime extractants and the reaction conditions which favor the formation of polynuclear copper complexes with dianionic forms of the reagents which could, consequently, enhance mass transport efficiency in recovery processes (Equation (2)).

nLH_2(org)_ + nCu^2+^ = [Cu(L)]_n(org)_ + 2nH^+^ (2)

**Figure 2:**
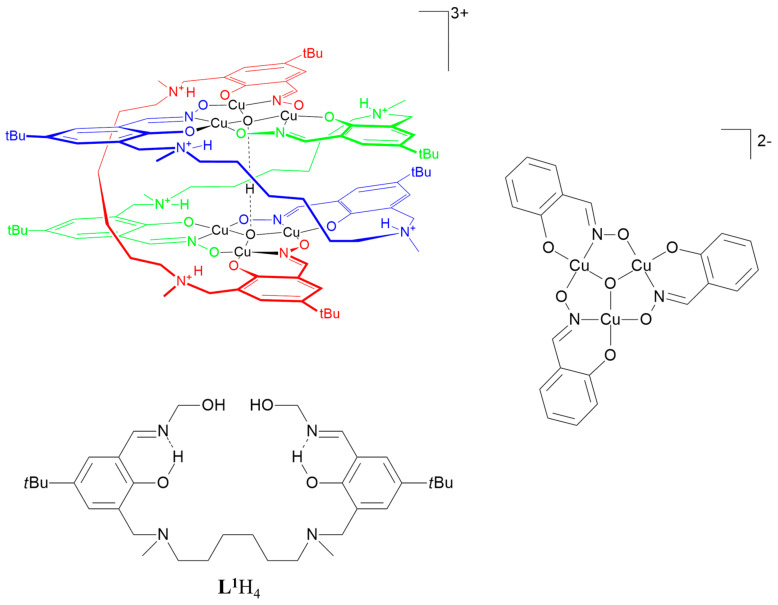
The structure of the hexanuclear complex [{Cu_3_O}_2_H(**L^1^**H_2_)_3_]^3−^ derived from **L^1^**H_4_ and a representation of the triangular cores [21].

There are few reports of copper(II) complexes of doubly deprotonated phenolic oximes. One describes a hexanuclear species [{Cu_3_O}_2_H(**L^1^**H_2_)_3_]^3+^ obtained from the proligand **L^1^**H_4_ which has two salicylaldoxime molecules linked by a [-CH_2_N(CH_3_)C_5_H_10_N(CH_3_)CH_2_-] unit [21]. In forming the complex, two protons are transferred to the amine N atoms in each ligand. The two trinuclear {Cu_3_-μ_3_-O}^4+^ units are held together by the three [-CH_2_NH(CH_3_)C_5_H_10_NH(CH_3_)CH_2_-]^2+^ straps and by a proton between the μ_3_-oxygen atoms as shown in Figure 2.

**Figure 3:**
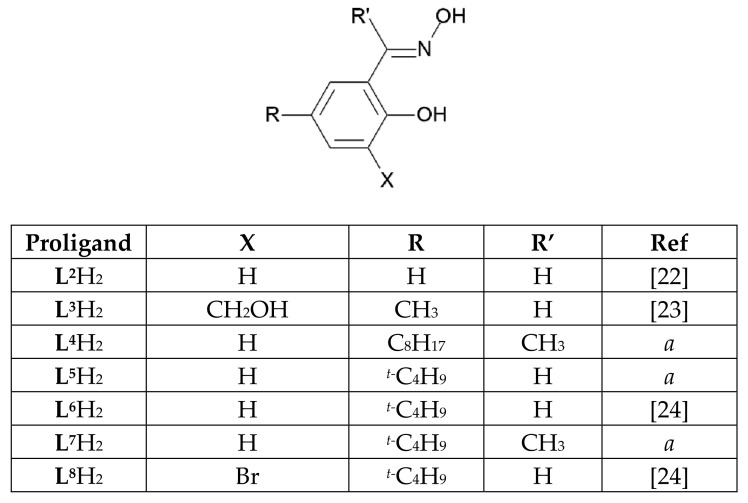

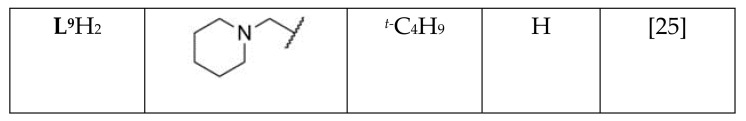
The phenolic oxime extractants and related proligands studied in this work. *a*—Details of synthesis and characterization can be found in the Experimental Section.

The motif of two co-facial Cu_3_O units linked by a proton in an O…H…O bridge has also been found in a complex containing the singly deprotonated form of unsubstituted salicylaldoxime, **L^2^**H_2_, in Figure 3 [22]. In this very complicated structure, the triangular Cu_3_O^2−^ units form stacks with short μ_3_-O…μ_3_-O distances (2.532(9) and 2.46(1) Å) being associated with the H-bridges. Much longer distances (7.047(9) and 7.31(1) Å) are found between the μ_3_-O atoms which do not carry a bridging proton.

**Figure 4:**
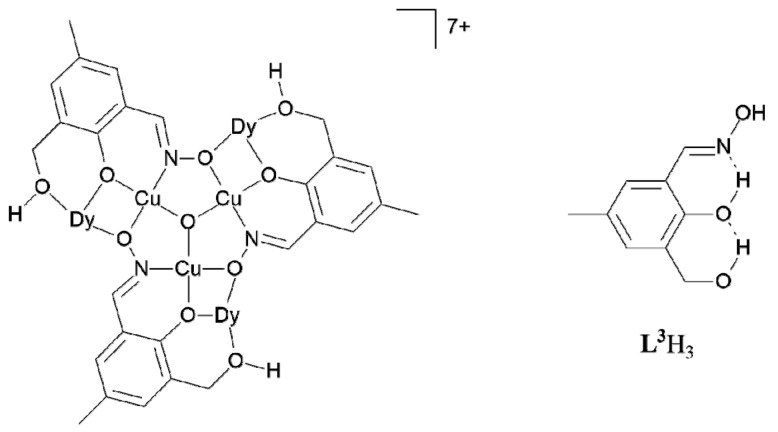
The [Cu_3_Dy_3_(**L^3^**H)O]^7+^ component of the heterometallic cluster [Cu_8_Dy_3_(**L^3^**H)_6_(μ_4_-O)_2_Cl_6_(H_2_O)_8_]Cl_8_ formed by the 2-hydroxymethyl-substituted phenolic oxime **L^3^**H_3_. All three Dy atoms are displaced to the same side of the Cu_3_ triangle [23].

Another Cu complex containing doubly deprotonated phenolic oximes and a Cu_3_O core is formed by **L^3^**H_3_ which contains a 2-hydroxymethylene substituent *ortho* to the phenolic O atom [23]. This makes it possible for rare earth trications to be incorporated on the edges of the Cu_3_O^2−^ triangles as shown in Figure 4. In this structure the 2-hydroxylmethylene is not deprotonated.

**Figure 5:**
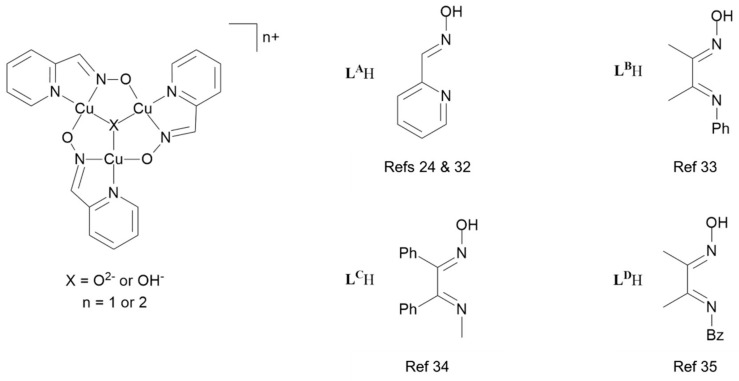
A representation of the Cu_3_O and Cu_3_OH cores present in complexes formed by 2-pyridinaldoxime, **L^A^**H [26,34], and some related α-imino-oximes (**L^B^**H–**L^D^**H) [35,36,37] which have only one ionizable H atom.

Deprotonated N-heterocycles with 2-aldoxime or 2-ketoxime substituents and α-imino-oximes (examples in Figure 4) form polynuclear Cu complexes with oximate bridges. Those derived from 2-pyridinaldoxime, **L^4^**H, were formulated as [Cu_3_**L^4^**_3_OH]X_2_ (X = ½ SO_4_^2−^, NO_3_^−^, ClO_4_^−^ and OH^−^, see Figure 5) [26]. When crystallized from solutions containing coordinating anions such as chloride or phosphonate, discrete trinuclear Cu(II) complexes are formed with the anion in axial sites on the Cu_3_O core [27,28,29]. Subsequently, many similar systems [30,31,32,33] have been shown to have hexanuclear structures with two co-facial Cu_3_O units linked by a proton in an O…H…O bridge.

Copper complexes formed by the proligands **L^A^**H–**L^D^**H in Figure 5 all have Cu_3_O or Cu_3_OH cores which contain the Cu atoms in fused 5-membered rings. In contrast, in the much smaller number of reported trinuclear Cu_3_O and Cu_3_OH complexes formed by phenolic oximes (Figure 3), each Cu atom is part of one 6-membered and two 5-membered rings.

Polynuclear oxo or hydroxy bridged copper units are also found in multicopper oxidases [24,38,39,40,41], but only a few examples of the proposed Cu_n_O_m_ active sites are thought to contain μ_3_-oxo or μ_3_-hydroxy bridged trinuclear units similar to the types which are formed oxime ligands listed in Figure 3 and Figure 5 which are the subject of this paper [42,43].

In this work, we set out to establish whether polynuclear Cu(II)-complexes can be generated from doubly deprotonated forms of the commercial phenolic oxime reagents. This would allow more efficient Cu extraction because they will have higher Cu(II) to extractant ratios, for example, as in Equation (2) above. The results are presented in a slightly unusual order, following the chronology of the experimental work. Analysis of the organic phase in solvent extraction experiments was first undertaken using electrospray ionization mass spectrometry (ESI:MS). The predominant anion present at high copper loading is a trinuclear Cu complex. Computational work, studies of electronic spectra, and X-ray structure determinations were then used to investigate the nature of the trinuclear complexes.

## 2. Results and Discussion

### 2.1. ESI:MS Studies

**Figure 6:**
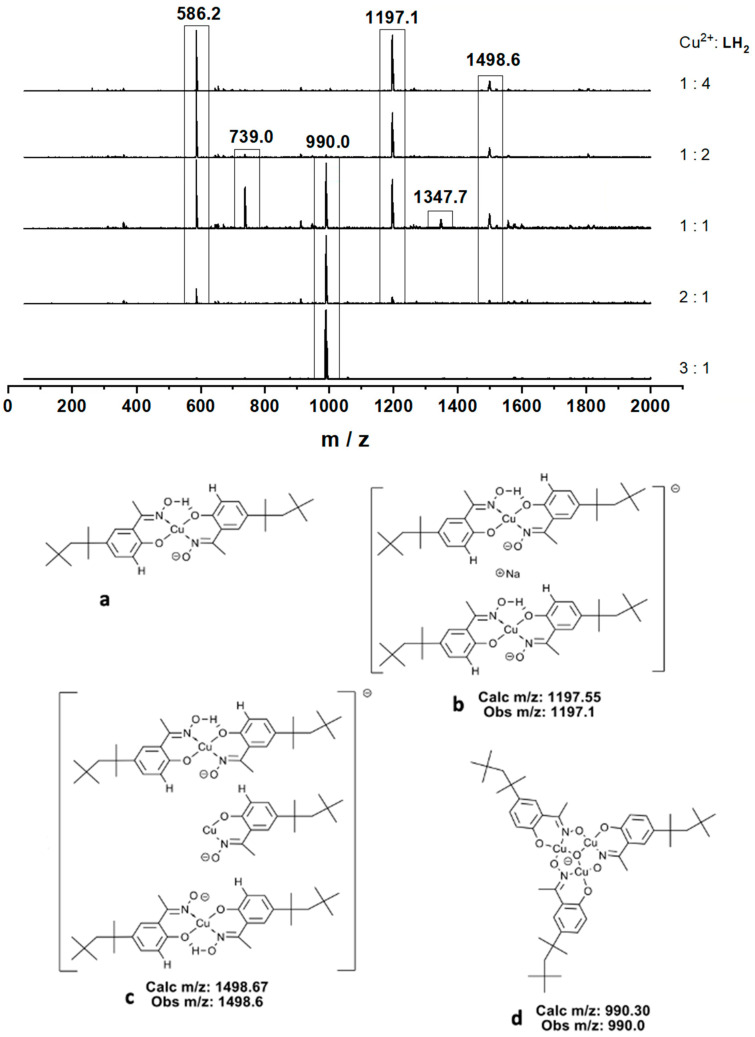
Suggested structures, together with their observed and calculated monoisotopic masses, for the dominant monoanionic species (**a**–**d**) in an acetonitrile solutions with different Cu(II):L^4^H_2_ molar ratios.

In biphasic solvent extraction systems, higher loading of Cu(II) by phenolic oxime reagents is favored by raising the pH of the aqueous phase and by operating extraction with high Cu(II):extractant ratios. In single-phase systems, the relative concentrations of Cu(II) complexes are not dependent on the relative solvation energies in the water and the water-immiscible phase, but for different ligands are still dependent on their pKa values and the formation constants of Cu(II) complexes. The speciation of a solution of **L^4^**H_2_ in MeCN (40 μM) with varying concentrations of Cu(OAc)_2_.H_2_O was monitored by electrospray ionization mass spectrometry (ESI-MS). Results are shown in Figure 6. When the Cu(II):**L^4^**H_2_ ratio exceeds 1:1, the dominant peak in the negative ion spectra has *m*/*z* 990.0 and can be assumed to have the trinuclear composition [Cu_3_O(**L^4^**)_3_]^−^ (see Figure 6d). At the highest molar ratio (3:1) this peak is 500 times more intense than any other in the spectrum.

Anions with the same composition, [Cu_3_O(L)_3_]^−^ (see Table 1), are also the dominant peaks in spectra of complexes formed in solution by a selection of the phenolic oxime proligands listed in Figure 3. Whilst it is likely that the relative intensities of species in the gas phase will not match the relative concentrations in solution, the order of energies from DFT calculations correlates with the observed relative intensities in the mass spectra.

At low molar ratios of Cu(II) to **L^4^**H_2_ (1:1, 1:2, and 1:4), the dominant peak (*m/z* 586.2, Figure 6a) is the mono-deprotonated form of the mononuclear complex [Cu(**L^7^**H)_2_], corresponding to the well documented structures formed by phenolic oxime extractants under normal operating conditions in Cu-recovery processes [4,5]. The intense peak at *m/z* 1197.6 observed in extracts at a Cu(II):**L^4^**H_2_ ratio of 1:2 (Figure 6b) can be assigned to an assembly containing two [Cu(**L^4^**)(L^4^H)]^−^ monoanions and a Na^+^ cation, and the peak at *m/z* 1498.6 (Figure 6c) to an assembly containing two [Cu(**L^4^**)(**L^4^**H)]^−^ monoanions and a [Cu(**L^7^**H)]^+^ cation, potentially linked via the deprotonated oximate oxygen and copper atoms. Other prominent peaks, for example at *m/z* 739.0 and 1347.7, can be assigned to other oligomeric species with the formulae ([Cu(**L^7^**)(**L^7^**H)] ([Cu(**L^7^**H)])*_n_*)^−^. There is no evidence for oligomeric species being formed in solution prior to ionization. Deprotonation of the neutral molecules [Cu(LH)_2_] at the interface to generate monoanions [Cu(LH)L]^−^ in the gas phase creates complexes with unfavorable repulsion between oximate oxygen atoms and adjacent coordinated phenolate oxygen atoms. This repulsion will be reduced by association with Cu(II) atoms in neighboring complexes as in **c** or with Na^+^ ions as in **b**.

**Figure 7:**
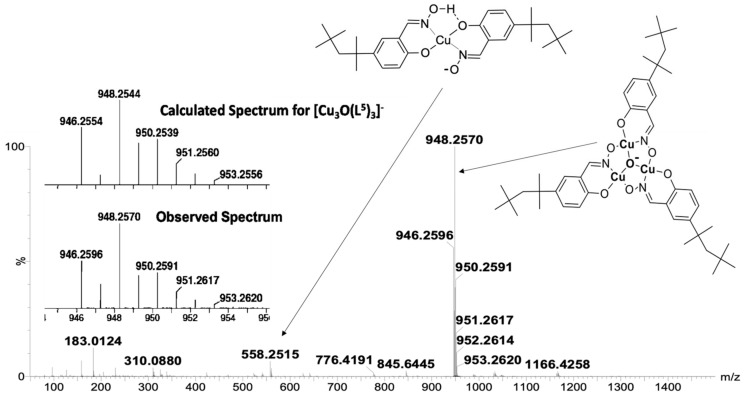
The high-resolution mass spectrum of a 50µM solution of **L^5^**H_2_ and copper acetate in acetonitrile. The observed and the calculated isotopic peak distribution and masses for [Cu_3_O(**L**^5^)_3_]^−^ are inset.

The formulation of [Cu_3_O(**L^4^**)_3_]^−^ as a trinuclear complex with a μ_3_-oxo unit rather than a μ_3_-hydroxo unit is supported by the high-resolution spectrum of [Cu_3_O(**L^5^**)_3_]^−^ obtained from a MeCN solution containing 50µM of **L^5^**H_2_ and a 1:3 Cu:**L^5^**H_2_ molar ratio (Figure 7). Formulation with an oxo bridge implies that one of the three copper atoms should be assigned a Cu(III) oxidation state in order to give the species a single negative charge. More realistically electrons in the Cu_3_O unit are likely to be delocalized over the three copper atoms. The [Cu_3_O(**L^5^**)_3_]^−^ moiety would be readily generated by heterolytic cleavage of the Cu_3_O…H…OCu_3_ bridges of the type found in hexanuclear assemblies formed by the monoanionic oximato ligands listed in Figure 5.

**Figure 8:**
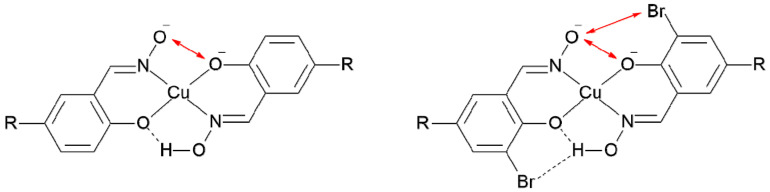
A representation of the origins of the stabilization of the monoanions [Cu(**L**)(**L**H)]^−^ by H-bonding in the lower part of the complexes (---) and of the destabilization by repulsion between electronegative atoms (double-headed red arrows in the upper part of the complexes).

The dominance of the peaks corresponding to anionic trinuclear complex [Cu_3_O(**L^5^**)_3_]^−^ in the ESI-MS is at first sight surprising, given that mononuclear 1:2 complexes, ([Cu(**L**H)_2_] see Figure 1) are the only species observed when the phenolic oximes are used in solvent extraction of copper [4,5]. The stability of these uncharged [Cu(**L**H)_2_] complexes is enhanced by their pseudo-macrocyclic structures involving the interligand H-bonding shown in Figure 1. However, if deprotonation of an oximic OH group occurs to generate the monoanionic species ([Cu(**L)**(**L**H)]^−^, the pseudo-macrocyclic structure is lost (see Figure 8) and the anion is destabilized by repulsion between the oximate and phenolate oxygen atoms [44].

**Figure 9:**
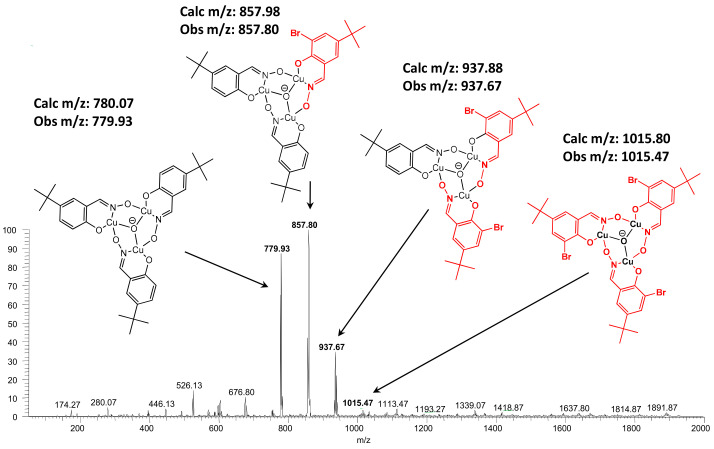
The mass spectrum obtained from a MeCN solution of Cu(II) acetate (50 µM) and **L^6^**H_2_ and **L^8^**H_2_ (each 25 µM). Proposed structures are inset with observed and calculated monoisotopic masses.

An MeCN solution containing a 2:1:1 molar ratio of copper(II) acetate and **L^6^**H_2_ and its 3-bromosubstituted derivative **L^8^**H_2_ gave an ESI-MS spectrum containing four trinuclear complexes: [Cu_3_O(**L^6^**)_3_]^−^, [Cu_3_O(**L^6^**)_2_(**L^8^**)]^−^, [Cu_3_O(**L^6^**)(**L^8^**)_2_]^−^, and [Cu_3_O(**L^8^**)_3_]^−^ (Figure 9). The relative intensities of these (0.9:1:0.4:0.02) in comparison to a homogenous statistical distribution (1:3:3:1) will depend on to what extent the presence of the bromine atom influences their relative stabilities and their intensities will also depend on their stabilities relative to the mononuclear complexes [Cu(**L^6^)**(**L^6^**H)]^−^, [Cu(**L^6^)**(**L^8^**H)]^−^, [Cu(**L^6^**H **)**(**L^8^**)]^−^, and [Cu(**L^8^)**(**L^8^**)]^−^ (the weak peaks clustered at *m/z* values at 447, 526, 526, and 605, respectively) which are formed on ionization of the neutral complexes [Cu(**L**H)_2_]. The stabilities of the monoanions are influenced by H-bonding and electrostatic repulsion as shown in Figure 8. The presence of the 3-bromo substituent buttresses the interligand H-bonding when the oxime OH group is present [45] but enhances repulsion between the ligands when the oxime proton is lost.t [45].

### 2.2. UV/Vis Spectra

**Figure 10:**
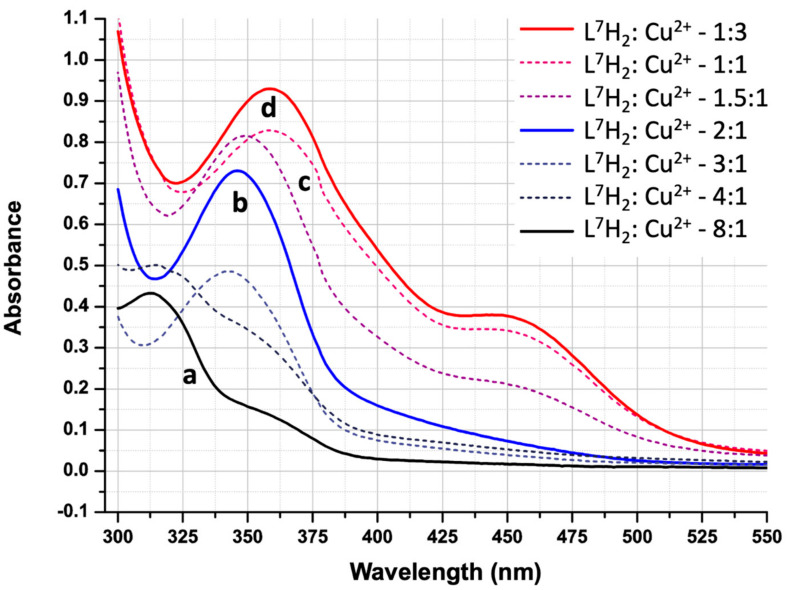
UV/Vis spectra of 80µM solutions of **L^7^**H**_2_** in MeCN containing varying concentrations of Cu(OAc)_2_.H_2_O (**L^7^**H**_2_**to Cu^2+^ molar ratio ranging from 1:3 to 8:1).

Variations in the UV/Vis spectrum of an 80 µM solution of **L^7^**H**_2_** in MeCN as the concentration of Cu(II) is increased were investigated to establish whether speciation is consistent with results obtained from mass spectra. Major changes in peak intensity and wavelength were observed (see Figure 10).

The proligand has an absorption maximum at 313 nm and a shoulder around 360 nm. When in excess, the tail of the proligand’s π-π* UV bands (Figure 10a) dominates the spectrum. Increasing the **L^7^**H**_2_**to Cu^2+^ molar ratio to 2:1 results in the π-π* maximum (Figure 10b) shifting to 346 nm, which can be assumed to arise from the increased conjugation found in the [Cu(**L^7^**H)_2_] complex. When the concentration of copper is equimolar to that of **L^7^**H**_2_** (Figure 10c) the π-π* maximum shifts to 359 nm and a second maximum at 446 nm is also observed. These bands do not shift position as the copper concentration is increased further (Figure 10d). The results are consistent with the speciation indicated by the ESI-MS results (above): only the 1:2 complexes [Cu(**L**H)_2_] or 1:1 trinuclear complexes containing either [Cu_3_O(**L**)_3_]^−^ or [Cu_3_OH(**L**)_3_] are present in significant concentrations in solution.

### 2.3. Solid State Samples and X-ray Structures of the Trinuclear Complexes

Despite the evidence that trinuclear complexes form readily in solution, particularly when the Cu to proligand molar ratio exceeds 1:1, it proved difficult to isolate crystalline samples of complexes of **L^2^**H**_2_**-**L**^8^H**_2_** suitable for X-ray structure determination. This contrasts with trinuclear complexes formed by α-imino-oximes (see Figure 5). Attempts to form crystalline trinuclear complexes of **L^2^**H**_2_**-**L^8^**H**_2_** using a variety of conditions including raising solution pH to promote formation of **L^2−^** dianions, adding monodentate ligands such as pyridine and piperidine to occupy axial sites and cations to charge-balance the [Cu_3_O(**L**)_3_]^2−^ unit were unsuccessful. Dark green, almost black, powders, very different in appearance from [Cu(**L**H)_2_] complexes, were obtained. Speculation that a girdle containing six anionic oxygen donor atoms might disfavor the isolation of crystalline trinuclear complexes is supported by the observation that crystalline complexes of the α-imino-oximes (Figure 5) have a girdle containing only three anionic oxygen donor atoms. It was reasoned that isolation of solid-state forms of polynuclear complexes containing bridging doubly deprotonated phenolic oximes might be more successful using ligands containing cationic groups adjacent to the phenolate O-atoms as this would reduce the negative charge density in the first and second coordination spheres of the Cu atoms in the [Cu_3_O(**L**)_3_]^2−^ units. This proposition was tested by preparing solutions containing equimolar quantities of the piperidinomethyl substituted proligand **L^9^**H**_2_**and Cu(II) acetate. These show mass and electronic spectra very similar to those observed for ligands with no 5-aminomethyl substituent. As with the negative ion spectra observed for complexes whose ligands do not contain the 5-aminomethyl substituent, the 2+ charge of the [Cu_3_O(**L^9^**H)_3_]^2+^ species implies that one of the three Cu-atoms should be assigned a Cu(III) oxidation state in order to balance the charge.

**Figure 11:**
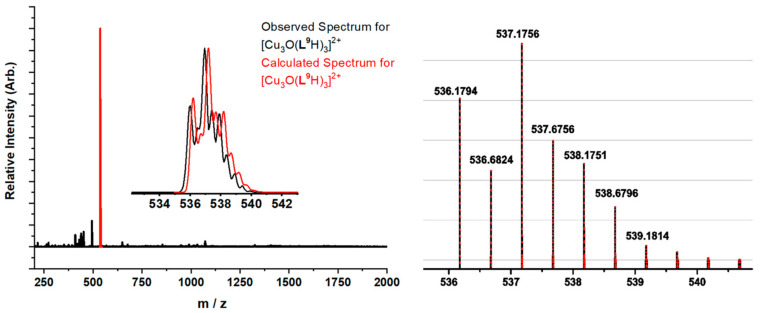
Low and high resolution (**left** and **right**) plots of the peak assigned to [Cu_3_O(L^9^H)_3_]^2+^ in a mass spectrum obtained from a 50 µM acetonitrile solution of [Cu_3_OH(**L^9^**H)_3_(ClO_4_)_2_]1.5H_2_O.

Evaporation of an acetonitrile solution yielded a green solid which was soluble in water. Addition of KClO_4_ gave a dark green precipitate with both low and high resolution mass spectra (Figure 11) suggesting that it contained the complex [Cu_3_O(**L^9^**H)_3_(ClO_4_)]^+^ in an assembly with a ClO_4_^−^ ion.

**Figure 12:**
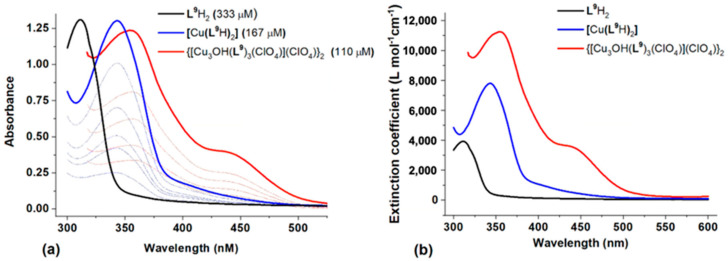
(**a**) The UV/Vis absorption spectra for varying concentrations of L^9^H_2_, [Cu(L^9^H)_2_] and {[Cu_3_OH(L^9^)_3_(ClO_4_)](ClO_4_)}_2_ and (**b**) their respective molar extinction coefficients, obtained by the absorbance vs. concentration plots form (**a**).

Optimization of reaction conditions generated a perchlorato complex in 91% yield which was assigned the hexanuclear formulation {[Cu_3_OH(**L^9^**H)_3_(ClO_4_)](ClO_4_)}_2_1.5H_2_O on the basis of microanalysis and the X-ray structure determination of two crystalline forms (see below). A comparison of the electronic spectrum of an acetonitrile solution of this material with those of **L^9^**H_2_ and [Cu(**L^9^**H)_2_] is shown in Figure 12. The variations in position of the absorption maxima are very similar to those observed for the proligand **L^7^**H_2_ (Figure 10) with a systematic red-shift of the π-π* band with increasing conjugation of the π system and a second absorption observed for the trinuclear unit [Cu_3_O(**L^9^**H)_3_(ClO_4_)]^+^ at 445 nm.

**Figure 13:**
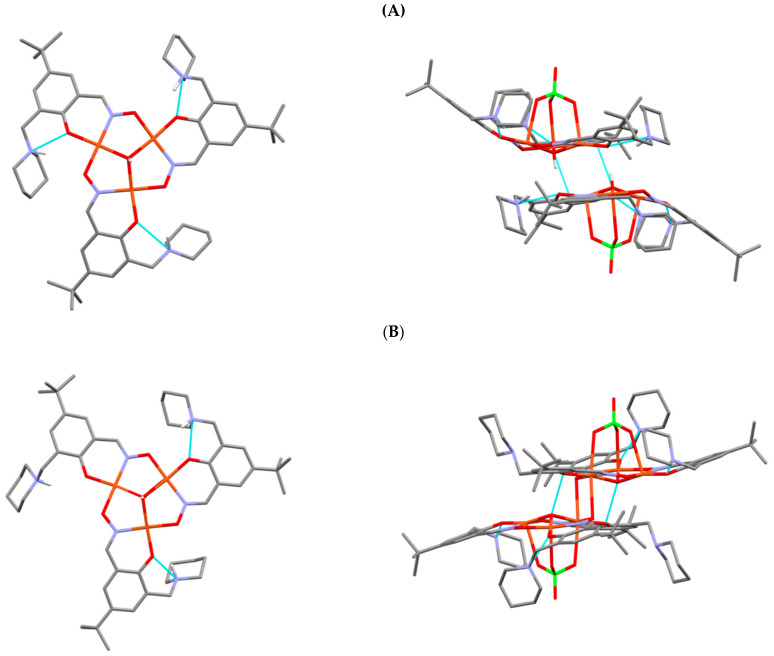
Components of the two crystalline forms (**A**,**B**) of the hexanuclear complexes **{**[Cu_3_OH(**L^9^**H)_3_(ClO_4_)](ClO_4_)**}**_2_.2H_2_O.2MeCN and **{**[Cu_3_OH(**L^9^**H)_3_(ClO_4_)](ClO_4_)**}**_2_.11H_2_O.MeCN. The trinuclear entities [Cu_3_OH(**L^9^**H)_3_]^2+^ and the hexanuclear units [Cu_3_OH(**L^9^**H)_3_(ClO_4_)]**_2_^2+^**are shown on the left and right, respectively. H-atoms not involved in H-bonding have been removed for clarity.

The high solubility of {[Cu_3_OH(**L^9^**H)_3_(ClO_4_)](ClO_4_)}_2_.2H_2_O.2MeCN in most polar organic solvents and in water and the facile separation the mononuclear complex [Cu(**L^9^**H)_2_] from non-polar solvents made isolation of the crystalline forms of the hexanuclear complex difficult. Two crystalline forms A and B were eventually obtained. Both contain trinuclear monocations [Cu_3_OH(**L^9^**H)_3_(ClO_4_)]^+^ with very similar structures (Figure 13) in which three oxygen atoms of a perchlorate ion occupy the axial sites of the copper atoms and the μ_3_-OH groups are displaced slightly to the other side of the Cu_3_ triangles.

**Figure 14:**
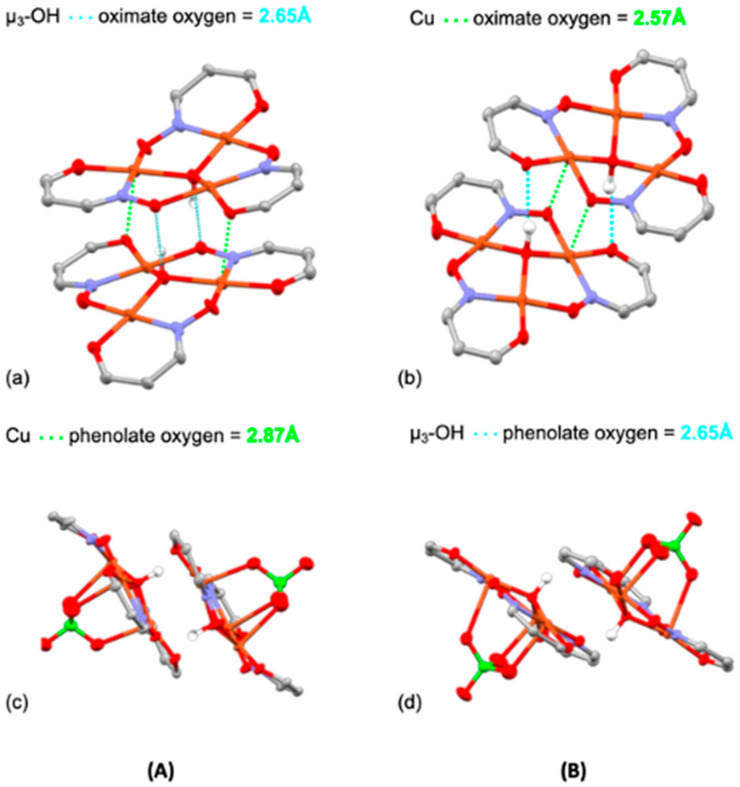
The central Cu-chelating units of forms (**A**,**B**) of the hexanuclear complexes {[Cu_3_OH(**L^9^**H)_3_(ClO_4_)](ClO_4_)}_2_.1.5H_2_O and {[Cu_3_OH(**L^9^**H)_3_(ClO_4_)](ClO_4_)}_2_.11H_2_O.MeCN showing the secondary bonding which creates the dimeric structures. The shortest Cu**^…^**O contact distances and the O**^…^**O distances associated with hydrogen bonds formed by the µ_3_-OH groups listed are marked. The pendant piperidinium groups have been removed for clarity.

The most significant difference between the crystalline forms is the way in which the trinuclear components assemble to generate the hexanuclear complex. Unlike most other hexanuclear Cu complexes, (see for example Figure 2), the two triangular units are not brought together by formation of a μ_3_-O^…^H^…^μ_3_-O bridge. In the crystalline form **A**, the two trinuclear units are held together by each μ_3_-OH group forming a hydrogen bond to an oximate O-atom (μ_3_-OH^…^O, 2.65 Å) in the adjacent unit and by two Cu-atoms forming weak bonds to a phenolate O-atom, as seen in Figure 14. In form **B** the intermolecular bonding involves each μ_3_-OH group forming a hydrogen bond to a phenolate O-atom (μ_3_-OH^…^O, 2.84 Å) in the adjacent unit and by two Cu-atoms forming weak bonds (2.573(2) Å) to an oximate O-atom. The very weak intermolecular Cu…phenolate bonds have lengths (2.871(3) and 2.573(2) Å in **A** and **B**) typical of those found in copper complexes formed by phenolic oximes [46].

The most significant difference between the crystalline forms is the way in which the trinuclear components assemble to generate the hexanuclear complex. Unlike most other hexanuclear Cu complexes, (see for example Figure 2), the two triangular units are not brought together by formation of a μ_3_-O^…^H^…^μ_3_-O bridge. In the crystalline form **A**, the two trinuclear units are held together by each μ_3_-OH group forming a hydrogen bond to an oximate O-atom (μ_3_-OH^…^O, 2.65 Å) in the adjacent unit and by two Cu-atoms forming weak bonds to a phenolate O-atom, as seen in Figure 14. In form **B** the intermolecular bonding involves each μ_3_-OH group forming a hydrogen bond to a phenolate O-atom (μ_3_-OH^…^O, 2.84 Å) in the adjacent unit and by two Cu-atoms forming weak bonds (2.573(2) Å) to an oximate O-atom. The very weak intermolecular Cu…phenolate bonds have lengths (2.871(3) and 2.573(2) Å in **A** and **B**) typical of those found in copper complexes formed by phenolic oximes [46].

The four donor atoms in the inner coordination spheres of the Cu(II) atoms are nearly planar (see Figure 13), as found in mononuclear Cu(II) complexes of phenolic oximes [46] and bond lengths fall in similar ranges. In both **A** and **B** the weak bonding commonly found [46] in axial sites of mononuclear Cu(II) complexes involves interactions with perchlorate counter anions.

The piperidinium nitrogen atoms in both crystalline forms **A** and **B** make close contact with neighboring phenolate oxygen atoms but the orientation of their NH^+^ groups suggest that H-bonding to lattice water molecules is a strong interaction. Quite different patterns of intermolecular H-bonding involving NH^+**…**^H_2_O units are found in the **A** and **B** forms. These and the crosslinking by water molecules to generate a polymeric structure.

In contrast to the solid-state structures discussed above, the predominant monoanionic species in the mass spectra appear to have µ_3_-oxo rather than µ_3_-hydroxo central bridges. The relative energies of various species are shown in Table 2 along with the deprotonation enthalpies of [Cu_3_(**L^2^**)_3_OH]^−^ and [[Cu_3_(**L^2^**)_3_OH] and the ionization enthalpies for [Cu_3_(**L^2^**)_3_OH]^−^ and [Cu_3_(**L^2^**)_3_O]^2−^.

The deprotonation of the anion [Cu_3_(**L^2^**)_3_OH]^−^ (**a**) to give the dianion [Cu_3_(**L^2^**)_3_O]^2−^ (**b**) is less favorable than that for the neutral complex [Cu_3_(**L^2^**)_3_OH] (**c**) to give the anion [Cu_3_(**L^2^**)_3_OH]^−^ (**d**). This is to be expected as it will be more difficult to increase the charge on the already negatively charged species.

**Figure 15:**
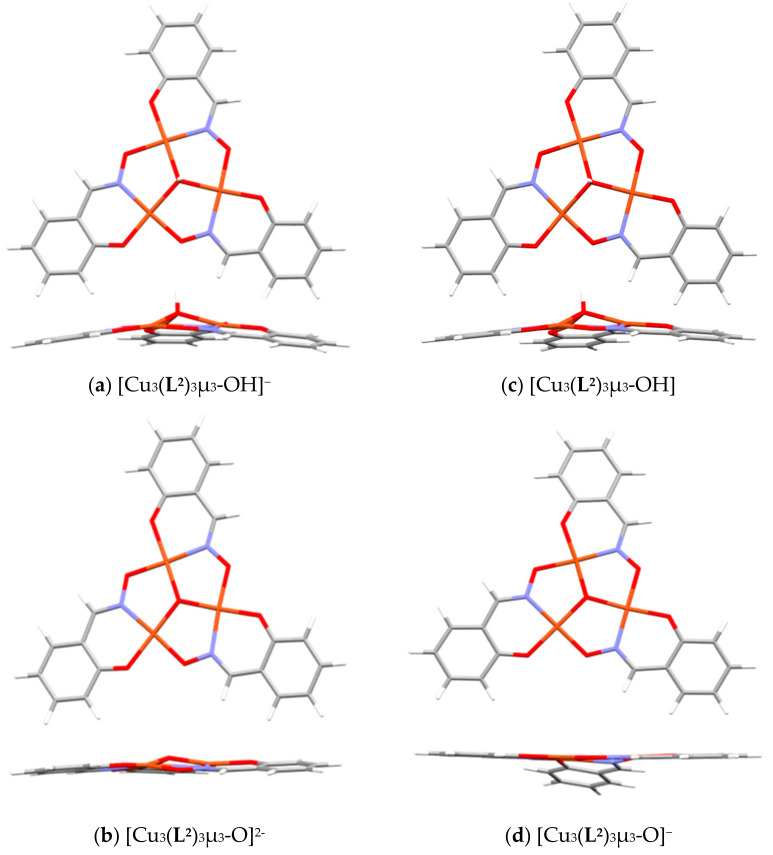
The energy-minimized structures for (**a**) [Cu_3_(**L^2^**)_3_μ_3_-OH]^−^, (**b**) [Cu_3_(**L^2^**)_3_μ_3_-OH], (**c**) [Cu_3_(**L^2^**)_3_μ_3_-O]^2−^ and (**d**) [Cu_3_(**L^2^**)_3_μ_3_-O]^−^.

Figure 15 shows the geometry-optimized structures for the four species described in Table 3.

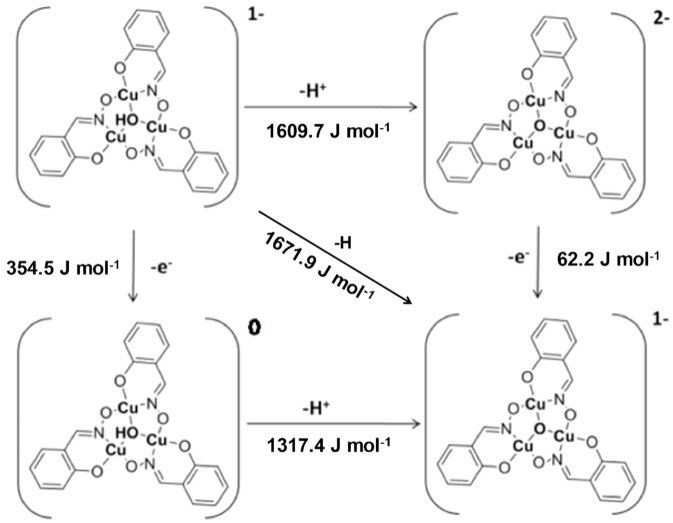



When the central unit is OH^−^, as in [Cu_3_(**L^2^**)_3_μ_3_-OH]^−^ or [Cu_3_(**L^2^**)_3_μ_3_-OH]**,** (Figure 15a,c)**,** the metallocycle unit is domed with the µ_3_-OH oxygen atom lying 0.560 and 0.432 Å respectively above the plane, defined by the three Cu atoms. When the central unit is O^2−^, Figure 15b,d, the core is nearly planar with deviation of the µ_3_-oxo atom of 0.052 and 0.002 Å from the Cu_3_ plane as might be expected by the sp^2^ hybridization of the former.

## 3. Materials and Methods

### 3.1. Materials and General Procedures

Unless otherwise mentioned, all solvents and reagents were used as received from Aldrich, Fisher, Fluorochem and Acros.

^1^H and ^13^C NMR were obtained using a Bruker AC250 spectrometer at ambient temperature. Chemical shifts (δ) are reported in parts per million (ppm) relative to internal standards.

All gas phase speciation studies using Electrospray Ionization Mass Spectrometry (ESI-MS). were carried out on single phase solutions of the a proligand, **L^x^**H_2_ (or a mix of multiple proligands) containing varying molar ratios of Cu(OAc)_2_.H_2_O (ACS Grade, Sigma Aldridge, St. Louis, MO, USA) in MeCN (HPLC Grade, Sigma Aldridge). A Thermo-Fisher LCQ mass spectrometer was used with a direct injection ESI source. All studies were carried out using the same experimental conditions detailed in Table 4. Each reported spectrum is an average of 250 scans extrapolated using Thermo-Fisher’s Xcalibur Qualbrowser 2.0 software and the results were then exported into Excel 2007.

High precision mass spectra were recorded on a Waters Quadrupole-Time-of-Flight (Q-ToF) spectrometer in conjunction with an Aquity Sample Manager and Binary Solvent Manager. All were obtained under identical conditions (Table 5). Each spectrum reported is an average of 20 scans of 4 independently diluted solutions.

X-ray diffraction data were collected at 150 K on a Bruker Apex-II diffractometer with Mo-Kα radiation (λ = 0.71073 Å). The data were corrected for absorption and other systematic effects using the multi-scan procedure SADABS. The structures were solved by direct methods (SHELXS) and refined by full-matrix least squares against |*F*|^2^ (SHELXL) [58]. All non-H atoms were refined with anisotropic displacement parameters unless otherwise noted below, with H-atoms placed in deal positions. In the case of water crystallization, H-atom placement was based on difference maps where possible or otherwise on favorable H-bond formation.

### 3.2. Synthesis of Proligands and Complexes

The syntheses of the proligands **L^1^**H_4_ [21], **L^6^**H_2_ [45], **L^8^**H_2_ [59], and **L^9^**H_2_ [60] have been reported previously. Details of the synthesis and characterization of the other proligands follow.

#### 3.2.1. 5-t-Octyl-2-Hydroxyphenylethanone Oxime (**L^4^**H_2_)

Acetyl chloride (86 g, 1.1 mol, 1.1 eq.) was added dropwise to a solution of 4-*t*-octylphenol (206 g, 1 mol, 1.0 eq.) in toluene (600 mL). The resulting solution was refluxed for 4 h and stirred overnight. Aluminum chloride (133 g, 1.0 mol, 1.0 eq.) was added in 48 portions to the stirred solution over 4 h and the mixture then heated under reflux for 3 h and stirred overnight at room temperature. The reaction was quenched with excess HCl (20%, 500 mL), added dropwise over 1 h. The organic phase was separated from the aqueous, washed with distilled water (2 × 250 mL), and filtered using phase separation paper. The solvent was removed in vacuo yielding a viscous yellow oil (230.2 g) 67% pure by gas chromatography. Hydroxylamine sulfate (221 g, 1.35 mol, 1.5 eq.) and sodium acetate (221 g, 2.7 mol, 3.0 eq.) were added to a solution of the crude methoxyketone (230 g, 0.9 mol, 1.0 eq) in ethanol (600 mL). The resulting suspension was refluxed for 2 h, allowed to cool, and poured onto toluene (500 mL) and distilled water (300 mL). The organic layer was separated, washed with distilled water (2 × 100 mL) and brine (100 mL) and filtered through phase separation paper. The solvent was removed in vacuo and the resulting yellow solid was purified via complexation with copper. The complex was washed with methanol (2 × 100 mL) and the resulting dark brown crystals were dissolved in toluene (500 mL). The copper was stripped from the toluene solution using sulfuric (0.1M, 3 × 200 mL), washed with distilled water (200 mL) and filtered through phase separation paper. The solvent was removed in vacuo and the solid was recrystallized from hexane yielding 5-*t*-octyl-2-hydroxyphenylethanone oxime as an off-white solid (135 g, 0.51 mol, 51% yield). Crystals suitable for XRD analysis were grown by slow cooling and evaporation of a saturated diethyl ether solution (details of the crystal structure analysis can be found in the Cambridge Structural Database (CSD) as ref code ABAWAD, having been previously published [45]. (Anal. Calc. for C_16_H_25_NO_2_: C, 72.96; H, 9.57; N, 5.32% Found: C, 73.0; H, 10.1; N, 5.5%; ν_max_/cm^−1^ (CHCl_3_) 3570 (free NOH), 3390br (H-bonded NOH), 3219br (PhOH), 2947 (C-H), 1602 (C=N); ^1^H NMR (400 MHz, CDCl_3_): δ_H_ (ppm) 0.75 (s, 9H, C(C*H*_3_)_3_), 1.39 (s, 6H, C(C*H*_3_)_2_), 1.76 (s, 2H, C*H*_2_), 2.41 (s, 3H,ArCNC*H*_3_) 6.93 (dd, 1H, Ar*H*), 7.31 (dd, 1H, Ar*H*), 7.42 (d, 1H, Ar*H*),11.10 (s, 1H, O*H*), 11.30 (s, 1H, O*H*); ^13^C NMR (400 MHz, CDCl_3_): δ_C_ (ppm) 10.90 (1C, ArCN(**C**H_3_)), 31.68 (2C, C(**C**H_3_)_2_), 31.84 (2C, C(**C**H_3_)_3_), 32.36 (1C, **C**(CH_3_)_3_) 38.02, (1C, **C**(CH_3_)_2_), 56.96 (1C, **C**H_2_), 116.57 (1C, aromatic **C**), 117.50 (1C, aromatic **C**H), 124.94 (1C, aromatic **C**H), 128.97 (1C, aromatic **C**H), 140.52 (1C, aromatic **C**), 155.14 (1C, Ar**C**N(CH_3_)), 155.1 (1C, aromatic **C**).

#### 3.2.2. 2-Hydroxy-5-t-Octylbenzaldehyde Oxime (L**^5^**H_2_)

Magnesium turnings (20 g, 800 mmol), methanol (373 mL), toluene (160 mL), and magnesium methoxide (a few drops of 8% w/w methanol solution) were refluxed until all the magnesium had dissolved and H_2_ evolution had ceased. 4-*t*-Octylphenol (268 g, 1.30 mol) was added and the mixture heated under reflux for 1 h. Toluene (333 mL) was added and the solvent removed under vacuum as the methanol/toluene azeotrope. A slurry of paraformaldehyde (120 g, 4.0 mol) in toluene (200 mL) was added slowly with continuous distillation and heated for a further 2 h. After cooling to room temperature, H_2_SO_4_ (20%, 800 mL) was added slowly with stirring and the mixture was warmed to 50 °C to dissolve all solids. The product was extracted with toluene (2 × 400 mL), washed with H_2_SO_4_ (10%, 2 × 150 mL) and water (150 mL), dried over MgSO_4_ and the solvent removed in vacuo. Purification by silica-60 wet flash column chromatography (2% ethyl acetate in hexane eluent) gave 2-hydroxy-5-*t*-octylbenzaldehyde (**A**) as an off white solid (196 g, 65%) ^1^H NMR (250 MHz, CDCl_3_): δ_H_ (ppm) 0.68 (s, 9H, C(C*H*_3_)_3_), 1.30 (s, 6H, C(C*H*_3_)_2_), 1.66 (s, 2H, C*H*_2_), 6.85 (d, 1H, Ar*H*), 7.42 (s, 1H, Ar*H*), 7.48 (dd, 1H, Ar*H*), 9.82 (s, 1H, ArC*H*O), 10.80 (s, 1H, ArO*H*); ^13^C NMR (63 MHz, CDCl_3_): δ_C_ (ppm) 30.4 (2C, C(**C**H_3_)_2_), 30.8 (3C, C(**C**H_3_)_3_), 31.3 (1C, **C**(CH_3_)_3_), 36.9 (1C, **C**(CH_3_)_2_), 55.6 (1C, **C**H_2_) 116.0 (1C, aromatic **C**H), 119.0 (1C, aromatic **C**), 129.5 (1C, aromatic **C**H), 134.4 (1C, aromatic **C**H), 140.8 (1C, aromatic **C**), 158.4 (1C, aromatic **C**), 195.8 (1C, Ar**C**HO).

Hydroxylamine hydrochloride (0.709 g, 10.2 mmol) and potassium hydroxide (0.674 g, 10.2 mmol) were dissolved separately in EtOH, mixed thoroughly and the white (KCl) precipitate removed by filtration. The filtrate was added to 2-hydroxy-5-*t*-octylbenzaldehyde (**A**, 2.120 ng, 9.1 mmol) and the mixture heated under reflux for 3 h. Evaporation of the solvent in vacuo gave a yellow oil, which solidified overnight. Recrystallization from hexane gave 2-hydroxy-5-*t*-octylbenzaldehyde oxime (**L^5^**H**_2_**) as white crystals (1.38 g, 60%). ^1^H NMR (250 MHz, CDCl_3_): δ_H_ (ppm) 0.64 (s, 9H, C(C*H*_3_)_3_), 1.23 (s, 6H, C(C*H*_3_)_2_), 1.58 (s, 2H, C*H*_2_), 6.80 (d, 1H, Ar*H*), 7.01 (s, 1H, Ar*H*), 7.20 (dd, 1H, Ar*H*), 8.12 (s, 1H, ArC*H*N); ^13^C NMR (63 MHz, CDCl_3_): δ_C_ (ppm) 31.2 (2C, C(**C**H_3_)_2_), 31.2 (2C, C(**C**H_3_)_3_), 32.7 (1C, **C**(CH_3_)_3_) 38.3, (1C, **C**(CH_3_)_2_), 57.3 (1C, **C**H_2_), 115.9 (1C, aromatic **C**), 116.4 (1C, aromatic **C**H), 128.5 (1C, aromatic **C**H), 129.9 (1C, aromatic **C**H), 141.9 (1C, aromatic **C**), 153.9 (1C, Ar**C**HN), 155.1 (1C, aromatic **C**).

#### 3.2.3. (5-t-Butyl-2-Hydroxyphenyl)Ethanone Oxime (**L^7^**H_2_)

Acetyl chloride (78.5 g, 1.00 mol, 1.1 eq.) was added dropwise to 4-*t*-butylphenol (135 g, 0.90 mol, 1.0 eq.) in toluene (600 mL). The resulting solution was refluxed for 4 h and stirred overnight. Aluminium chloride (120 g, 0.90 mol, 1.0 eq.) was added in 48 portions to the stirred solution of ester over 4 h. The mixture was heated under reflux for 3 h and then stirred overnight at room temperature before quenching with excess HCl (20%, 500 mL) which was added dropwise over 1h. The organic phase was collected, washed with distilled water (2 × 250 mL) and filtered through phase separation paper. The solvent was removed in vacuo yielding a viscous orange oil (190 g) 69% pure by GC. Hydroxylamine sulfate (221 g, 1.35 mol, 1.5 eq.) and sodium acetate (221 g, 2.7 mol, 3.0 eq.) were added to a solution of the crude methylketone (230 g, 0.90 mol, 1.00 eq.) in ethanol (600 mL) and the resulting mixture was heated under reflux for 2h, allowed to cool, and poured onto toluene (500 mL) and distilled water (300 mL). After stirring the organic layer was separated, washed with distilled water (2 × 100 mL) and brine (100 mL) and filtered through phase separation paper. The solvent was removed in vacuo and the resulting orange solid was recrystallized from heptane yielding 5-*t*-butyl-2-hydroxyphenyl)ethenone as an off white powder (119 g, 0.57 mol, 70% yield). Crystals suitable for XRD analysis were grown by slow cooling and evaporation of a saturated hexane/diethyl ether solution. The structure contains ethanol of solvation, which was treated with the SQUEEZE procedure (180 e per unit cell) [61]. Details of the crystal structure analysis can be found in the CDS as YUPCUJ, deposition number 1410150 [62]. Anal. Calc. for C_12_H_17_NO_2_: C, 69.54; H, 8.27; N, 6.76% Found: C, 68.9; H, 8.6; N, 6.4%; ν_max_/cm^−1^ (CHCl_3_) 3573 (free NOH), 3400br (H-bonded NOH), 3219br (PhOH), 2960 (C-H), 1620 (C=N); ^1^H NMR (400 MHz, CDCl_3_): δ_H_ (ppm) 1.32 (s, 9H, C(C*H*_3_)_3_), 2.41(s, 3H, ArCNC*H*_3_), 6.95 (d, 1H, Ar*H*), 7.38 (dd, 1H, Ar*H*), 7.45 (d, 1H, Ar*H*); ^13^C NMR (63 MHz, CDCl_3_): δ_C_ (ppm) 10.84 (1C, ArCN**C**H_3_) 31.52 (3C, C(**C**H_3_)_3_), 31.61, (1C, **C**(CH_3_)_3_), 116.82 (1C, aromatic **C**), 117.72 (1C, aromatic **C**H), 124.07 (1C, aromatic **C**H), 128.09 (1C, aromatic **C**H), 141.75 (1C, aromatic **C**), 155.17 (1C, aromatic **C**); 159.86 (1C, Ar**C**HN).

### 3.3. Syntheses of Complexes

Solutions of Cu(II)-complexes of the phenolic oximes **L^2^**H_2_-**L^9^**H_2_ were prepared under the conditions described in Section 2 and the speciation in solution was investigated using the spectroscopic techniques discussed in that section. The synthesis of solid state forms of two mononuclear Cu(II)-complexes, [Cu(**L^5^**H)_2_] and [Cu(**L^8^**H)_2_], was undertaken to generate complexes which were fully characterized and then redissolved to confirm that the speciation in solution matched that of the complexes prepared in situ. Stoichiometric amounts of the proligand and Cu(II) acetate (0.5 equivalents) were stirred in methanol (50 mL) for 24 h. Color changes due to complex formation occurred rapidly, along with precipitation. Complexes were isolated by filtration and dried under vacuum. The syntheses of [Cu(**L^4^**H)_2_] and [Cu(**L^4^**H)_2_DMSO] and their crystal structures (CDS codes BAYTOM and BAYTUS) have been reported previously [63]. The coordination geometry of the mononuclear complexes in this paper is unremarkable and for [Cu(**L^4^**H)_2_] and [Cu(**L^4^**H)_2_DMSO], (CDS codes BAYTOM and BAYTUS) has been reported and discussed previously [53]. These structures closely resemble those of other Cu(II) complexes of phenolic oximes, having very nearly planar N_2_O_2_^2−^donor sets and very weak axial interactions to donor atoms in neighboring molecules [46].

#### 3.3.1. [Cu(L**^5^**H)_2_]

Cu(OAc)_2_.H_2_O (40.0 mg, 0.20 mmol) and **L^5^**H_2_ (99.5 mg, 0.40 mmol) yielded a brown powder (103.5 mg, 93%). Anal. Calc. for C_30_H_44_O_4_N_2_Cu: C, 64.32; H, 7.92; N, 5.00. Found: C, 64.3; H, 8.0; N, 5.1%.

#### 3.3.2. [Cu(L**^8^**H)_2_]

Cu(OAc)_2_.H_2_O (0.100 g, 0.50 mmol) and **L^8^**H_2_ (0.270 g, 1.01 mmol) yielded a brown solid (0.284 g, 88%). Anal. Calc. for C_22_H_26_Br_2_O_4_N_2_Cu: C, 43.62; H, 4.33; N, 4.62. Found: C, 43.9; H, 4.1; N, 4.5%.

#### 3.3.3. [Cu_3_OH(L**^9^**H)_3_(ClO_4_)_2_]1.5H_2_O

A solution of Cu(OAc)_2_.H_2_O (0.2 g, 1 mmol, 1.0 Eq.) in MeCN (20 mL) was added to a stirred solution of triethylamine (1.5 mL, 5 mmol, 5 Eq.) and **L^9^**H_2_ (0.290 g, 0.1 mmol) in MeCN (20 mL). The resulting dark green solution was stirred for 30 min at room temperature and the solvent was removed in vacuo yielding a dark green crystalline solid. This was redissolved in distilled water (20 mL) and lithium perchlorate (0.52 g, 5 mmol, 5 Eq.) in distilled water (10 mL) was added. The suspension was stirred for 30 min and filtered, yielding [Cu_3_OH(**L^9^**H)_3_(ClO_4_)_2_]1.5H_2_O as a dark green powder. (0.39 g, 91%). Anal. Calc. for: C_102_H_152_N_12_O_33_Cl_4_Cu_6_ C, 47.17; H, 5.90; N, 6.47. Found: C, 47.1; H, 5.9; N, 6.4; ESIMS *m/z* 537.46 [(Cu)_3_(**L^9^**)_3_µ_3_-O]^2+^. Crystals of {[Cu_3_OH(L**^9^**H)_3_(ClO_4_)](ClO_4_)}_2_.2H_2_O.2MeCN for X-ray structure determination were obtained by slow cooling a saturated solution in MeCN:H_2_O:ethyl acetate, 5:1:4. All components in {[Cu_3_OH(L**^9^**H)_3_(ClO_4_)](ClO_4_)}_2_.2H_2_O. 2MeCN occupy general positions. The piperidine moiety of the **L^9^**H ligand based on O1B is disordered over two orientations, and the same comment applies to the MeCN of solvation. Anisotropic refinement of the oxygen atoms of the perchlorate counter-anion developed large and irregular displacement parameters, a sign of unresolved disorder, and these atoms are modelled isotropically in the final model. CCDC deposition 2,181,162 contains the supplementary crystallographic data for this structure.

Triclinic crystals of {[Cu_3_OH(**L^9^**H)_3_(ClO_4_)](ClO_4_)}_2_.11H_2_O.MeCN were obtained by slow diffusion of a solution of [Cu_3_OH(**L^9^**H)_3_(ClO_4_)_2_]1.5H_2_O in a 1:1 mixture of MeCN and diethyl ether into H_2_O. In {[Cu_3_OH(**L^9^**H)_3_(ClO_4_)](ClO_4_)}_2_.11H_2_O.3MeCN the hexanuclear Cu complex resides on an inversion center. The *t*-Bu group attached to the **L^9^**H ligand based on O1C is disordered over two orientations. Disorder is likely to be present in the ligand based on O1B in both the *t*-Bu and piperidine groups, but this was modeled with prolate anisotropic displacement parameters. The uncoordinated perchlorate counter-anion is disordered over two positions and orientations. The asymmetric unit also contains are five fully occupied and one half-occupied molecules of water of crystallization together with one fully occupied and one half-occupied molecules of MeCN. Structural parameters for this structure have been previously deposited on the CSD and are available as ref code YUPCIX (deposition number 1410143).

## 4. Conclusions

We have shown that phenolic oximes of the type widely used as solvent extractants in Cu recovery can form complexes with higher Cu to extractant molar ratios than the 2:1 value involved in current industrial processes [4].

2**L**H_2(org)_ + Cu^2+^ = [Cu(**L**H)_2_]_(org)_ + 2H^+^ (Cu:**L** molar ratio 1:2)

This could increase the efficiency of Cu recovery because a given inventory of extractant in the circuit will transport more Cu. Lower extractant to Cu molar ratios arise when the phenolic oxime is doubly deprotonated and loading equations such as

n**L**H_2(org)_ + nCu^2+^ = [Cu(**L**)]_n(org)_ + 2nH^+^ (Cu:**L** molar ratio 1:1)

become possible. In practice, we have shown that when double deprotonation of phenolic ketoximes and aldoximes takes place in solution, the formation of trinuclear complexes with the generic formula [Cu_3_(**L^2^**)_3_μ_3_-X]^n−^, where X is OH^−^ or O^2−^, is favored.

An unconventional sequence of research activities proved to be effective in this work; ESI-MS indicated that in solution polynuclear complexes of the types previously only commonly observed [7−15] for trivalent transition metal ions can also be formed with Cu(II) and demonstrated that the formation of such Cu complexes is favored by raising the pH and the concentration of Cu relative to oxime. This information helped define conditions to isolate solid-state samples of Cu complexes containing trinuclear moieties. In practice, it was difficult to isolate crystalline samples suitable for X-ray structure analysis, and this only proved possible in the specific case with (i) the piperidino substituted proligand **L^9^**H**_2_**when supramolecular interactions led to the formation of hexanuclear assemblies {[Cu_3_OH(**L^9^**H)_3_(ClO_4_)](ClO_4_)}_2_ in which the central μ_3_-OH group formed H-bonds to either oximate or phenolate oxygen atoms in an adjacent trinuclear unit, and (ii) when perchlorate was present to cap the other sides lying on the pseudo 3-fold axes, forming weak bonds in all the apical sites of the Cu atoms.

Whilst this study confirms that the copper to phenolic oxime ratio can be increased by doubly deprotonating the extractant, it reveals that the conditions needed to achieve this make it unlikely that more efficient solvent extraction processes could be developed for commercial operation. Raising pH to doubly deprotonate the extractant will give rise to the precipitation of any Fe(III) in the feed solution which presents major operating problems. A key features of the current (low pH) processes using phenolic oximes is that they can treat pregnant leach solutions because they show high selectivity of extraction of Cu(II) over Fe(III) which is often present in excess [4,5].

## Figures and Tables

**Table 1:** The most intense anionic peaks detected in the ESI-MS spectra of MeCN solutions of Cu(OAc)_2_.H_2_O and a selection of the proligands in Figure 3.

L^n^H_2_	L^n^H_2_:Cu^2+^	Monoisotopic *m/z* for [Cu_3_(L)_3_O]^−^	Calculated Monoisotopic *m/z* for [Cu_3_(L)_3_O]^−^	Observed Intensity (%)
**L**^4^H_2_	1:3	990.01	990.30	100
**L**^5^H_2_	1:2	948.49	948.25	100
**L**^6^H_2_	1:2	779.93	780.07	100
**L**^7^H_2_	1:2	822.02	822.11	100
**L**^8^H_2_	1:2	1015.47	1015.80	100

**Table 2:** Ranges of bond lengths/Å and angles/^o^ in the inner coordination spheres of the Cu atoms in the two crystalline form **A** and **B**.

	Cu-N_oximate_	Cu-O_phenolate_	Cu-O_oximate_	Cu-O_hydroxide_
Bond lengths in **A**	1.949(3)–1.970(3)	1.872(3)–1.921(2)	1.906(3)–1.963(2)	1.932(3)–1.962(3)
and in **B**	1.941(2)–1.956(2)	1.882(2)–1.9207(19)	1.915(2)–1.9443(19)	1.950(2)–1.9791(19)
	N_oximate_-Cu-O_phenolate_	O_phenolate_-Cu-O_oximate_	O_oximate_-Cu-O_hydroxide_	O_hydroxide_-Cu-N_oximate_
Bond angles in **A**	92.16(12)–93.94(11)	85.07(11)–88.04(11)	89.82(11)–91.38(10)	89.72(11)–93.94(11)
and in **B**	93.18(10)–94.95(9)	84.72(8)–86.07(8)	87.99(8)–91.57(8)	88.75(9)–91.05(9)

**Table 3:** The relative enthalpic energies of the µ_3_-hydroxo complexes (a) [Cu_3_(**L^2^**)_3_OH]^−^ and (b) [Cu_3_(**L^2^**)_3_OH] and the µ_3_-oxo complexes (c) [Cu_3_(**L^2^**)_3_O]^2−^ and (d) [Cu_3_(**L^2^**)_3_O]^−^ and the calculated deprotonation enthalpies of [Cu_3_(**L^2^**)_3_OH]^−^ and [[Cu_3_(**L^2^**)_3_OH] and ionization enthalpies/kJ mol^−1^ for [Cu_3_(**L^2^**)_3_OH]^−^ and [Cu_3_(**L^2^**)_3_O]^2−^. Geometry optimizations used the Gaussian ’03 program with the level of theory B3LYP [47,48] 6-31+G [25,49,50,51,52,53,54,55,56,57] (d,p).

**Complex**	(**a**) [Cu_3_(**L**^2^)_3_OH]^−^	(**b**) [Cu_3_(**L**^2^)_3_OH]	(**c**) [Cu_3_(**L**^2^)_3_O]^2−^	(**d**) [Cu_3_(**L**^2^)_3_O]^−^
**Charge**	−1	0	−2	−1
**Multiplicity**	4	3	4	3
**Enthalpy (Hartree)**	−6421.9645	−6421.8295	−6421.3514	−6421.3277
**Enthalpy (J mol^−1^)**	−16,860,868	−16,860,513	−16,859,258	−16,859,196
**E**_deprotonation_ [Cu_3_(L^2^)_3_OH]^−^ (a) → [Cu_3_(L^2^)_3_O]^2−^ (c)				1610
**E**_deprotonation_ [Cu_3_(L^2^)_3_OH] (b) → [Cu_3_(L^2^)_3_O]^−^ (d)				1317
**E**_ionisation_ [Cu_3_(L^2^)_3_OH]^−^ (a) → [Cu_3_(L^2^)_3_OH]^2−^ (b)				355
**E**_ionisation_ [Cu_3_(L^2^)_3_O]^2−^ (c) → [Cu_3_(L^2^)_3_O]^−^ (d)				62

**Table 4:** Machine settings employed for the Thermo-Fisher LCQ mass spectrometer.

Sample Flow Rate	5 µL/min
Sheath gas flow rate	46 (Arb. Units)
Aux. gas flow rate	0 (Arb. Units)
Capillary temperature	190 °C
RF amplitude	560 V
Spray Voltage	4.5 kV
Capillary Voltage	−26.5 V
Octapole 1 offset	6.8 V
Lens Voltage	13 V
Octapole 2 offset	10.5 V
Tube lens offset	0 V

**Table 5:** Machine settings employed for the Waters Q-ToF mass spectrometer.

Sample Injection Volume	6 µL
Desolvation gas flow rate	800 L/h
Aux. gas flow rate	30 L/h
Source temperature	90 °C
Desolvation Temperature	350 °C
Spray Voltage	2.2 kV
Extraction Cone	4.0
Sampling Cone	80
Ion Guide	2.0

## Data Availability

Crystallographic data have been deposited with the Cambridge Crystallographic Data (see experimental section).
